# Correction

**DOI:** 10.1080/15476286.2025.2449742

**Published:** 2025-01-07

**Article title**: Relevance of RNA to the therapeutic efficacy of mesenchymal stromal/stem cells extracellular vesicles

**Authors**: T., Teck Ta and S., Kiang Lim

**Journal: *RNA Biology***

**Bibliometrics**: Volume 22, Number 01, pages 01 - 07

**DOI**: https://doi.org/10.1080/15476286.2024.2446868

This article was published earlier with an error in the caption of figures. This has now been corrected and republished in the original article.

Figure 1a. EV publications on therapy and diagnostics

Total number of publications on EVs (PubMed search query: ‘extracellular vesicles OR exosomes’) is 64,215. 21800 (34%) were on therapy and diagnostics, respectively (PubMed search query: ’(extracellular vesicles OR exosomes) AND therapy’. 17621 (27%) were on diagnosis (PubMed search query: (extracellular vesicles OR exosomes) AND diagnosis”).

Figure 1b. Publications on EVs and types of RNA

Of 64,215 publications on EVs 19,053 (30%) were on RNA (PubMed search query: ‘RNA AND (extracellular vesicles OR exosomes)“), 10754 (56%) on microRNAs (PubMed search query: ”(Extracellular vesicles OR microvesicles OR exosomes) AND microRNA’), 3,743 (20%) on mRNA (PubMed search query: ‘RNA AND (extracellular vesicles OR exosomes) AND (mRNA OR messenger RNA)“), 736 (4%) were on long noncoding RNAs (PubMed search query: ”(Extracellular vesicles OR microvesicles OR exosomes) AND long noncoding RNA’), and 1,030 (5%) were on circular RNAs (search query: ‘RNA AND (extracellular vesicles OR exosomes) AND (circular RNA)’).

Figure 1c. Publications on EVs and RNA for therapy and diagnostics

Of the 19,053 publications on RNA and EVs, 6,150 (32%) were on therapy (PubMed search query: ‘RNA AND (extracellular vesicles OR exosomes) AND therapy’) and 5,310 (28%) on diagnosis (PubMed search query: ‘RNA AND (extracellular vesicles OR exosomes) AND diagnosis’).

Figure 1d. Cell source of EV RNA for therapy

The main cell source of 6,150 publications in Figure 1c on EVs or exosomes is stem cells, constituting 1949 or 32% of the publications (PubMed search query: RNA AND (extracellular vesicles OR exosomes) AND therapy AND stem cell’). Of these 1949 publications, 1,305 publications (67%) were on MSCs (PubMed search query: ’RNA AND (extracellular vesicles OR exosomes) AND therapy AND stem cell AND (MSC OR mesenchymal stromal cell)”.

Figure 1e. The limitations of an RNA-based hypothesis for MSC-EV therapeutic activity

RNA within MSC-derived EVs predominantly consists of fragments smaller than 300–500 nucleotides. Given that the average length of mRNA in a mammalian cell is approximately 2200 nucleotides, mRNA present in EVs is likely fragmented and thus non-functional. EV-associated miRNAs represent only 2–5% of the total small RNA population, with estimates suggesting that even the most abundant miRNA exists at fewer than one copy per EV. This is in stark contrast to mammalian cells, where miRNAs constitute around 80% of the small RNA pool with copy numbers as high as > 100 000 per cell for the most abundant miRNAs. Consequently, miRNAs are underrepresented in EVs and found at low concentrations. Additionally, the uptake of EVs by recipient cells is limited, with less than 1% being internalized within one hour and only about 30% of internalized EVs released into the cytoplasm. This implies that the amount of EV miRNA delivered into recipient cells is insufficient to elicit any significant biological or pharmacological response. Collectively, these findings suggest that mRNA and miRNA in EVs are unlikely to elicit substantial biological activity.

